# Developing and testing a toolkit of interventions to improve adherence to non-invasive ventilation in children: the SPIRITUS study protocol

**DOI:** 10.1136/bmjopen-2024-090570

**Published:** 2024-11-28

**Authors:** Jessica Russell, Elaine Y Chan, Gwyneth Davies, Faith Gibson, Philip Harniess, Garry Rendle, Jo Wray

**Affiliations:** 1 Centre for Outcomes and Experience Research in Children's Health, Illness and Disability (ORCHID), Great Ormond Street Hospital for Children NHS Foundation Trust, London, UK; 2 Pediatrics, Great Ormond Street Hospital for Children NHS Foundation Trust, London, UK; 3 UCL Great Ormond Street Institute of Child Health Library, London, UK; 4 School of Health Sciences, University of Surrey, Guildford, UK; 5 Heart and Lung Directorate, Great Ormond Street Hospital for Children NHS Foundation Trust, London, UK

**Keywords:** respiratory medicine (see thoracic medicine), sleep medicine, feasibility studies

## Abstract

**Abstract:**

**Introduction:**

Non-invasive ventilation (NIV) is a known effective and safe treatment for children and young people with sleep disordered breathing (SDB). Adherence can be challenging and poor adherence risks undertreatment of SDB. While the risk factors for non-adherence have been widely reported, very few interventions have been tested in any capacity to address barriers to adherence.

**Methods and analysis:**

We will conduct a mixed methods study over three phases. The aim is to identify the components of a toolkit of interventions to address some of the barriers to NIV usage in children and young people who have SDB. We will test these components for their feasibility and acceptability to families. We will also aim to identify health outcomes from NIV use that are important to families. Qualitative data will be managed using NVivo software and analysed using the Framework method. Quantitative data will be analysed using descriptive statistics.

**Ethics and dissemination:**

The study will run from January 2023 to October 2025. This study has ethics and local site approval. Data will be stored and accessed only by the research team, stored on a secure server. Data will be pseudo-anonymised prior to analysis and presented in a way that no individual can be identified. We will disseminate widely to relevant stakeholders including peer-reviewed journals, presentations to academic and clinical audiences at conferences and meetings and a lay report.

**Trial registration number:**

ISRCTN56845190.

**STRENGTHS AND LIMITATIONS OF THIS STUDY**

- Our study site has one of the largest patient cohorts of paediatric non-invasive ventilation (NIV) users in the UK, thereby allowing us to recruit sufficient numbers of children and families
- Recruitment will include a clinically heterogenous group, encompassing all the major condition groups where NIV use is likely.
- We have included a comprehensive approach to patient and public involvement, with parent advisors embedded within our study steering committee and children’s and young people’s involvement through Great Ormond Street Hospital’s Young People’s Advisory Group.
- We will use a range of data collection methods, including digital and creative approaches, to create an inclusive research environment for a population with a high rate of comorbidities who may find traditional methods alone difficult to access.
- Selection bias may occur as a result of parents having to consent for their child’s participation; we will use a sampling matrix to mitigate this risk.
- This is a single-site study; results of this study will need to be piloted on a larger scale with multiple sites.

## Introduction

 Long-term non-invasive ventilation (NIV), including continuous positive airway pressure (CPAP) and bilevel positive airway pressure (BiPAP), is a common form of breathing support delivered by a ventilator via an interface (a mask). It is used for a wide range of sleep-related breathing disorders resulting from upper airway obstruction, neuromuscular/musculoskeletal disorders, pulmonary conditions, obesity and central nervous system disorders in children, young people and adults.

The child patient population is growing as indications for NIV expand. In a recent scoping review,^1^ NIV use was reported for 73 conditions. According to Wilkinson *et al*,^2^ almost 1700 children in the UK use domiciliary NIV. The population of children and young people who use NIV is clinically heterogenous and is known to be medically complex. A multicentre retrospective cohort study^3^ found that almost all children and young people had at least one additional comorbidity, 25% were supported by additional technologies and only 16% discontinued NIV due to improvement, highlighting the long-term nature of support required. In the last decade, while their use of hospital services has remained high, care for these children has shifted from hospital to home.^4^ Consequently, parents are taking on increasing levels of responsibility for their child’s healthcare, including NIV care.

Sleep disordered breathing (SDB)—the disruption of normal respiratory patterns and ventilation during sleep—has multiple implications for children’s health. Chronic, untreated SDB is associated with hypertension, cardiovascular disease, metabolic disorders and neuropsychiatric and developmental issues.^5^ NIV has been shown to increase survival of children with neuromuscular disorders who are most vulnerable to respiratory morbidities, with improved survival attributed to ventilator support.^6^ Neonates/Infants with upper airway obstruction,^7^ such as those with craniofacial and neurological/neuromuscular conditions,^8^ form a significant group of NIV users. Children with SDB can present with disturbed sleep, nocturnal enuresis and confusional arousals, resulting in behavioural problems including attention deficit and hyperactivity disorder.^9^

Despite the heterogeneity of the cohort, 73% of studies in a scoping review^1^ concluded that long-term use of NIV provides benefits, including health, developmental, educational and psychosocial improvements, with better neurocognitive and behavioural outcomes.^10 11^ Improving or resolving SDB can also lead to benefits in quality of life, such as increased energy and a greater willingness to participate in activities.^12^

However, adherence to NIV can be challenging and cause significant disruption to families.^13^ From our previous study (the NIV Adherence study), we found that parents report the highest levels of treatment and condition burden when adherence is poor, along with high rates of parent overnight vigilance.^14^ For the child, adverse events associated with NIV include facial side effects,^15^ mask intolerance, leak or NIV therapy intolerance, nasal symptoms, device failure and abdominal distension,^1^ all of which can negatively impact adherence. Bedi *et al*^16^ found that as the number of complications from NIV usage increased, the odds of continuing to use NIV decreased by 8.7%. Adherence is therefore varied and often suboptimal. Watach *et al*^17^ found that for 1079 cumulative participants across 20 studies, positive airway pressure was adhered to by <60% of participants, with an average usage of 4.0–5.2 hours per night. A sizeable number of children are at potential risk of undertreatment with potentially adverse consequences.

Levert *et al* emphasised the need for a socioecological framework to assess the barriers to the implementation of NIV, with evidence-based intervention strategies.^18^ Since no single intervention will remove all barriers, we need a range of options that health professionals can use flexibly and tailor to the individual needs of children and young people and their families. We therefore want to develop a toolkit of educational, behavioural, organisational and/or environmental interventions to increase adherence rates so that children and young people do not experience extended periods without NIV use.

### Aims and research questions

#### Aim 1

To identify a range of theoretically informed educational, behavioural, organisational and/or environmental interventions for a toolkit to support children and young people requiring NIV and their families.

##### Research questions

- What interventions exist to address adherence to NIV in children and young people?
- How effective are these identified interventions for improving children’s and young people’s NIV adherence?
- In what context has improved adherence occurred?

### Aim 2

To test the identified interventions to assess their feasibility and acceptability.

#### Research questions

- Are identified interventions acceptable to parents and children and young people?
- Are identified interventions feasible for parents and children and young people?

### Outcome measures

#### Aim 3

To identify salient outcomes of NIV use and their metrics for children and young people and their families.

#### Research questions

- What outcomes do staff, parents and children and young people identify as important to measure the impact of NIV use?
- What metrics should be used to measure these outcomes?

### Patient and public involvement

Two parents from the study steering committee of our previous study (the NIV Adherence study) have provided feedback to the research team on all aspects of the study to date, including design and data collection methods. A further three to four parents have been invited to participate in the same role. The study steering committee will also include healthcare professionals from Great Ormond Street Hospital and other centres and representatives from a company that makes the ventilators. Feedback from children and young people from the Great Ormond Street Hospital’s Young People’s Advisory Group has informed the design of this study, ensuring that our approach is accessible, inclusive and family focused.

### Research Ethics Committee and other regulatory review and reports

The study has been reviewed and approved by the West Midlands-Solihull Research Ethics Committee (ref: 23/EM/0102). It has also been registered with and approved by the Research and Innovation Office at Great Ormond Street Hospital for Children NHS Trust, who will act as study sponsor.

## Methods

In Phases I and II, a realist review and workshops will occur in an iterative co-design process where one informs the other to identify the components of the toolkit of interventions. In Phase III, the toolkit of interventions will be tested for feasibility and acceptability.

### Rapid realist review

A realist approach to evaluation will be used, known as a ‘realist review’, focusing on understanding causal relationships/interactions between an intervention programme’s mechanisms (M) and context (C) to produce outcomes (O) within original research studies. These findings are synthesised and modelled within a context-mechanism-outcome configuration to answer the question ‘what works for whom and in what circumstances?’

We will apply rapid analytical methods as outlined by Saul *et al*^19^ and will follow the method for analysis outlined by Pawson *et al.*^20^,^20^ The RAMESES (Realist And Meta-narrative Evidence Syntheses: Evolving Standards) publication standards^21^ will be used for reporting results of the realist review.

Objectives and focus of review: the review will address the following questions:

1. What interventions exist to facilitate adherence to NIV in children and young people (aged 0–18 years)?
2. What interventions exist to facilitate adherence to NIV in adults (aged 18+ years)?
3. What specific barriers do the interventions identified in^1^ seek to address?

#### Search strategy

Development of search terms: we will collaboratively identify terms likely to be relevant to the project scope, purpose and research questions. We will identify articles and documents for inclusion in the review (both from published and grey literature) identified by knowledge users and content experts. In addition, we will use the search terms to iteratively generate lists of documents that may be included in the review. Initial search terms will be piloted to inform the final search strategy.

#### Selection criteria

*Inclusion criteria*: all study designs and healthcare settings, articles or sources of evidence that mention interventions aimed at improving adherence to NIV in children and young people (aged 0–18 years) and/or adults (aged 18+ years), articles published from 1980 (the invention of CPAP) to the present date.

*Exclusion criteria*: articles that describe NIV use in children or adults for palliative care, NIV use for children or adults who are medically dependent on NIV (eg, conditions such as congenital central hypoventilation system), articles not written in English, written before 1980 and/or those in which major quality concerns are raised during the critical appraisal process, study protocols.

*Databases*: Medline, PsychINFO, EMBASE, CINAHL, Emcare, Web of Science, SCOPUS, Cochrane.

*Grey literature*: stakeholders will be asked to recommend information sources.

#### Screening process

Results from database searches will be entered into Covidence software,^22^ which will be used to remove duplicate articles, collate and screen bibliographic records. Two researchers will independently screen all article titles and abstracts and full-text review will be undertaken for any article selected by one or both researchers. Full-text review will also be undertaken independently by two researchers and a third researcher will be available if consensus about inclusion in the review cannot be reached.

#### Data extraction

A bespoke data extraction form will be used to extract the following information from included articles: source details, study design, participant information (age, sex, diagnoses) interventions that address NIV and outcomes of the intervention(s).

#### Synthesis

Researchers will synthesise extracted data in rounds of iterative stages. Results of the initial searches will be presented in workshops held with healthcare professionals, stakeholders, parents and children and young people. Interventions that are raised in the first round of workshops that have not been identified in the literature will result in new targeted literature searches. Subsequent rounds of literature searches will inform the second round of workshops, where participants will vote on the components of the final toolkit.

Researchers and members of the study steering committee will discuss the interventions to ascertain: (1) how an intervention works (i.e. how the programme’s infrastructure and resources trigger particular decisions or behaviours in human participants, resulting in specific outcomes) and (2) whether interventions can be grouped.

This team-based approach will establish preliminary programme theories for each intervention, in the form of causal statements that identify enabling or constraining factor(s), the impact of those factors and the outcomes that resulted, typically in a ‘if…then…’ statement structure. These theories will be tested on a sample of the literature and the group will meet again to refine the programme theories. Any changes that are made to the review process will be documented.

### Workshops (phases I and II)

#### Inclusion criteria

Children and young people, hereafter referred to as ‘children’, who meet the following criteria will be eligible to participate in the study: age 0–18 years, have a diagnosis of SDB, have been prescribed NIV >3 months prior to recruitment and are under the care of the study site NIV team. Children will be ineligible to participate if they are medically dependent on NIV, receive NIV for palliation, have used NIV for <3 months and/or are in the process of weaning off NIV. NIV is defined as ventilatory assistance through a non-invasive interface (ie, not an endotracheal tube or a tracheostomy). It comprises CPAP and BiPAP.^23^

Parents or carers (adults with parental responsibility) of eligible children will be eligible to participate, unless they are members of the SPIRITUS study steering committee.

Staff at the study site who provide care for children who use NIV (medical, nursing, allied health professionals, healthcare assistants, sleep-unit technicians, physiologists) will be eligible to participate.

#### Recruitment process

Children will be identified from a large single paediatric centre (Great Ormond Street Hospital for Children (GOSH)). To ensure that a representative sample of NIV users is included, purposive sampling using a sampling matrix will be used to ensure diversity in terms of the child’s age, sex, ethnicity, underlying health condition, duration of NIV use, level of adherence to NIV (high adherence is defined as >4 hours use per night for >70% of nights and low adherence is defined as <4 hours use per night for >70% of nights^24^) and presence or absence of a learning disability. In situations where parents or children report that they or their child identifies differently from their birth-assigned sex, we will respect their preferred gender. We will aim for an equal distribution of participation across these variables. To ensure parity, we have established upper and lower parameters for each variable in the matrix.

#### Invitation approach

We will aim to recruit 8–12 parent-child dyads, where both the parent and the child are participants. All participants over 16 years of age will be asked to provide consent for their participation. Children under the age of 16 will be asked to provide assent for their participation. Parents can participate irrespective of their child’s participation and children can participate with or without their parent, if their parent provides consent for them to do so.

We will also aim to recruit 8–10 staff members.

All parent and staff participants will be asked to participate again in Phase II; children will only be asked to participate in Phase I. Where attrition occurs between Phases I and II, we will aim to recruit new parent and/or staff participants for phase II.

### Parents and children

All eligible families will be introduced to the study by a NIV member of staff at the study site. Families will be approached to participate at their NIV outpatient appointment/NIV sleep study (in person or digital appointment) or another GOSH appointment. When these options are not available, a cover letter will be sent by the clinical team with information sheets and consent forms enclosed, followed by a phone call by a member of the study site staff to check that the information has been received and to address any questions the family may have.

### Staff

An ‘all-user’ email to potential staff participants will be sent by the lead of the NIV service offering the opportunity to participate in a group workshop, which may be face-to-face or online.

### Data collection

#### Phase I

During Phase I, we will facilitate a series of workshops and/or individual interviews using creative methods (such as Talking Mats) and informed by topic guides. Separate workshops will be held with children who use NIV and their parents, and staff groups across a range of disciplines (medical, nursing and allied health professionals) and representatives from industries that manufacture ventilator equipment for children. Where children have learning disabilities or additional needs, researchers will work with families to ascertain the most appropriate means of enabling the child to express themselves, including working with their parents as communication partners. Interpreters will be available to facilitate the inclusion of non-English-speaking children and parents on an as-needed basis. Discussions will be framed around the elements of the Behaviour Change Wheel.^25^ This is a theoretical framework for understanding how behaviours change as a result of an intervention, identifying whether current and proposed interventions impact the three major domains of motivation, capacity and opportunity. Consent will be sought to audio record workshops.

In the workshops, participants will be invited to discuss interventions that impact adherence and NIV outcomes identified from two sources: findings from a previously completed study at our centre^14^ and the results from the preliminary extraction and synthesis from the realist review, as well as any interventions that participants themselves have identified.

Participants will also be asked to identify any short-term, medium-term or long-term outcomes that they would like to see as a result of treatment.

#### Phase II

In Phase II, there will be a second round of workshops, in which participants will discuss interventions identified in Phase I and from revised literature searches. We will use the nominal group technique^26^ to generate, record, discuss and vote on ideas and rounds of voting will enable us to finalise a list of interventions to be included in the toolkit together with a list of outcomes.

We will offer families a range of participation options to fit with their lives caring for their child, including both group-based workshops and one-to-one sessions with a researcher, which can be held in person or digitally.

Workshops and interviews from Phases I and II will be audio-recorded with consent and transcribed verbatim.

### Qualitative data analysis

Qualitative data from Phases I and II will be managed using NVivo. Transcripts will be analysed using the Framework approach,^27^ an analytical process involving five distinct but highly interconnected stages: (1) familiarisation; (2) identifying a thematic framework; (3) indexing; (4) charting; (5) mapping and interpretation. Frameworks developed for Phases I and II will be used to identify barriers to NIV use, existing interventions to overcome these barriers, interventions participants would like to see implemented and outcomes of NIV as identified by participants.

#### Phase III

##### Development of the intervention toolkit

We will discuss the results of the workshops with the study steering committee to explore the practicality, accessibility and feasibility of each intervention. Intervention development will be informed by a range of patient and public involvement (PPI) approaches including parent members of the study steering committee and members of the Young People’s Advisory Group. Some interventions may be resources, others may involve therapeutic interactions with a particular clinical team. Some interventions may already exist in another form, others may need to be adapted from other medical specialisms or yet to be developed.

For all interventions, we will identify the tasks required to complete the development of each intervention, working with our appropriate partners (e.g. industry) and feasible timelines for the creation of any new resources. Where it is not possible to design and create the intervention during the course of the study, a prototype of that intervention will be developed and tested.

### Feasibility and acceptability testing of the intervention toolkit

#### Inclusion criteria

Children aged 0–18 years who have a diagnosis of SDB, have been prescribed NIV for >3 months prior to recruitment and are under the care of the study site NIV team will be eligible to participate. Children will be ineligible to participate if they are medically dependent on NIV, receive NIV for palliation, have used NIV for <3 months or are in the process of weaning off NIV.

Parents or carers (adults with parental responsibility) of eligible children will be able to take part, unless they are members of the SPIRITUS study steering committee, took part in Phases I and/or II or were involved in the PPI activities around intervention development. Each parent-child dyad will be able to participate in hase III once.

In Phase III, we will aim to recruit 10–12 parent-child dyads. These sample sizes are in line with other similar feasibility/acceptability studies; they will provide an insight into how acceptable both the interventions in the toolkit and the data collection methods used in the study are to families.^28 29^ Unlike in Phases I and II, parent-child dyads will not be able to participate independently. Parents who wish to participate must also consent for their child’s participation and children cannot take part if their parent does not also consent to participate.

#### Recruitment

The sampling strategy for Phase III will be the same as Phases I and II but will additionally include the choice of intervention (ie, we will increase our invitation rates if participants choose the same interventions as previous participants).

All eligible families will be introduced to the study by a NIV staff member at the study site. Families will be approached to participate when they come for their NIV outpatient appointment/NIV sleep study (in person or digital appointment) or another GOSH appointment. When these options are not available, a letter will be sent with information sheets and consent forms, followed by a phone call by a study site member of staff to check that the information has been received and to ask if the family have any questions.

#### Data collection

In Phase III, we will assess acceptability (using the toolkit) and feasibility (implementing the toolkit in practice) and identify outcomes with children and families.

Participating families will be invited to discuss their particular challenges with NIV and their desired outcomes from an intervention with the clinical nurse specialist during their routine clinical appointments or over the phone. The researcher will liaise with the clinical nurse specialist prior to the consultation as to whether they want to introduce the toolkit at the beginning or end of the consultation; the timing of the introduction will not have an impact on the study outcome.

In face-to-face/virtual appointments, the clinical nurse specialist will show and describe the contents of the toolkit and invite families to discuss each intervention using a ‘Think Aloud’^30^ approach and describe their thoughts and feelings of their own situation. Families will be asked to choose the intervention they want to test and identify which outcome they hope the intervention will impact, for example, school attendance, reduction in night-time waking, etc. They will be asked to test the intervention for 2 weeks. The researcher will observe and document the chosen intervention, outcome and decision-making in field notes.

It is likely that clinical appointments with families who have lower levels of adherence will be more likely to result in a recommended clinical change (eg, new interface, changed pressure settings, introduction of a new comfort measure, etc) compared with families who do not report any issues and use NIV consistently. Families who have a recommended clinical change will be asked to start testing the intervention 2 weeks after the introduced change; families with no clinical changes will be asked to start immediately.

After the initial consultation, the researcher will collect baseline data related to the intervention and outcome from families.

Once home, during the 2-week test period, parents and children will be asked to record their experience of using the intervention, including the duration and any challenges they faced. They will be asked to wear an Actiwatch or equivalent device overnight during the 2-week trial period (children only) and keep an Actigraphy diary (older children/parent) to record data about sleep patterns and routines (eg, time child went to bed).

After 2 weeks, the researcher will visit the family at their home and ask the parents and, where possible and appropriate, the child to participate in a short semi-structured reflective interview (a debrief) about whether they used their chosen intervention as anticipated (fidelity to the intervention), any particular challenges, whether it was acceptable in practice, how feasible it was to use and perceptions of the outcome measurement. Children will be offered a range of creative methods to describe their experiences, such as Talking Mats^31^ or Draw and Write. Parents and, where possible, children will be asked to complete a postintervention questionnaire on the outcome and intervention. The researcher will collect all equipment and download the ventilator data onto an encrypted USB drive. Actiwatch data in conjunction with the ventilator data will enable us to identify whether the child: (1) used their NIV during the 2-week period and (2) if they used their NIV when asleep. This will mitigate potential response bias, for example, if parents report that the intervention resulted in complete adherence and the ventilator data show that the ventilator was not used in the study period. These data will also be collected to assess whether the intervention has caused a decrease in adherence.

If families are unable to find a time when a researcher can visit their home within the 2–4 weeks period, the researcher will offer the family a phone call or virtual meeting to complete data collection activities and make arrangements for the download of ventilator data and the return of study equipment (Actiwatch and iPad).

All discussions and interviews will be audio-recorded with families’ permission and transcribed verbatim for subsequent analysis. Demographic data and clinical information will be extracted from medical records for both analysis and reporting purposes.

### Data analysis

#### Qualitative analysis

Qualitative analysis in Phase III will follow the Framework Analysis steps outlined in Phases I and II. The Phase III framework will seek to identify participants’ (positive and negative) experiences of using the intervention during the 2-week period, how the parent and child engaged with it, what (if any) impact it had on their child’s NIV use and the wider family household and if the intervention should be changed in any way. Parent entries in their diaries will be used to inform both the postintervention debrief as well as analysed in their own right and included in the Phase III frameworks.

#### Quantitative analysis

We will compare usage data (number of hours used per night and number of nights used) during the 2-week trial period and usage data from the 4 weeks prior to the most recent ventilator download (usually in the NIV clinic or at the most recent sleep study). Questionnaire data will be analysed using descriptive statistics and recruitment rates, retention and fidelity to the intervention will also be analysed descriptively.

### Study outcomes

Increasing numbers of children are being prescribed NIV and we know that adherence is a problem for some children, placing these children at risk of suboptimal or undertreatment. We hope to provide a series of potential solutions to some of the barriers to non-adherence in this population by developing a toolkit of interventions that can be tailored to the individual child. At the end of hase III, we will have a toolkit of interventions and outcome measures ready to test for efficacy in a subsequent pilot study, for which funding will be applied for separately. In the longer term, we hope to improve adherence to NIV, with resulting improvements in health, educational and behavioural outcomes.

## Ethical approval

The study has been reviewed and approved by the West Midlands-Solihull Research Ethics Committee using the standard UK IRAS/HRA (Integrated Research Application System/Health Research Authority) application process. It has been registered with and approved by the Research and Innovation Office at Great Ormond Street Hospital for Children NHS Trust, who will act as the study sponsor (study number 21HL08). Additionally, SPIRITUS is listed on the ISRCTN registry with study registration number ISRCTN56845190.
