# Supplementary material for: Developing and testing a toolkit of interventions to improve adherence to non-invasive ventilation in children: the SPIRITUS study protocol
Source: BMJ Open. 2024 Nov 28;14(11):e090570. doi: 10.1136/bmjopen-2024-090570 (PMC11660321; doi:10.1136/bmjopen-2024-090570)
Supplement: Supplementary file 1 [file bmjopen-2024-090570.reviewer_comments.pdf]

PEER REVIEW HISTORY

BMJ Open publishes all reviews undertaken for accepted manuscripts. Reviewers are asked to complete a checklist review form and are provided with free text boxes to elaborate on their assessment. These free text comments are reproduced below.

ARTICLE DETAILS

Title (Provisional)

Developing and testing a toolkit of interventions to improve adherence to non-invasive ventilation in children: a mixed methods study (The SPIRITUS study)

Authors

Russell, Jessica; Chan, Elaine Y; Davies, Gwyneth; Gibson, Faith; Harniess, Philip; Rendle, Garry; Wray, Jo

---

VERSION 1 - REVIEW

**Reviewer**	**1**
**Name**	**Castro Codesal, Maria**
**Affiliation**	**University of Alberta, Pediatrics**
**Date**	**23-Nov-2023**
**COI**	**None**

Dear authors,

Thank you for putting together this important study. Please find my comments below to improve the clarity of your manuscript.

GENERAL COMMENTS

- The authors have written a thorough study protocol that is appropriate for a thesis proposal or grant application. However, further work is needed to adjust to scientific manuscript standards. Overall, it is a lengthy manuscript with significant amount of detail perhaps not always required while lacking some of the important information required to help the reader understand the purpose of the study and methods proposed. I have provided some examples below in comments to specific sections.

- Eliminating item lists will improve the clarity of the manuscript and shorten it significantly.

- Tables can also be eliminated and key information about inclusion criteria and ethics added to the manuscript in short paragraphs.

- It becomes sometimes redundant to have reference to phase 1, 2 and 3 several times through the text. I forces the reader to scroll up and down to find information about each

phase in different parts of the manuscript. I wonder whether it would be more efficient use of the space and easier to read if authors create a section for each phase of the project that includes all pertinent information: realistic review (search strategy, screening process, data extraction and synthesis); workshops 1 & 2 (inclusion criteria, recruitment process, methods, data synthesis and analysis); and phase 3 (recruitment, data collection, and data analysis).

- I struggled with the acronyms used through the manuscript, some of them not spelled out on the first time they were used. There is a number of non-conventional acronyms (CYP, SSC, YPAG, CNS.) that are hard to memorize and retain in reader's mind through the manuscript reading. Consider revising all acronyms and only leave the essential ones.

- There are a few format issues that might require authors to review author guidelines. For instance, abstracts and summaries do not include references, figures or tables.

- Authors have included references to unpublished studies and conference abstracts that do not meet authors guidelines.

SUMMARY:

Strengths section: I would argue that having a largest cohort is not a strength per se. Facilitation of adequate recruitment numbers or allowing for input from specialized healthcare providers with expertise in the use of NIV in pediatrics might. Having a large cohort can also be a limitation as it is because the study recruitment single center, as authors highlighted. Consider deleting it. The study have other methodological strengths including the high level of patient/family engagement and the use of multi-methods qualitative/quantitative that can be highlighted.

- Spell out GOSH

- It is unclear to the reader what a traditional methods for data collection is. Suggest to rephrase towards highlighting innovate methods for qualitative data collection based on the use of new technologies.

INTRO

- Sentence 'almost 1700 children in the United Kingdom (UK) use domiciliary NIV

' according to xxx [please provide the source].

- SDB and OSA are used in the introduction, and it is unclear to the reader if they are interchangeable. Consider selecting one of the terms or describing how they are different. As well, it will help the reader to understand if NIV refers to CPAP and BPAP both from the introduction.

- References needs to be revised. For instance, sentence 'For these children there are few viable treatments to resolve their SDB : long-term NIV may be their only option (-needs a reference). CYP with sub-optimally treated SDB are at greater risk of chest infections and repeat hospitalizations (reference 14 only applies to patients with NMD and not to patients with OSA and other comorbidities)

.- The last three paragraphs of the introduction could be more succinct and perhaps use more recent references. Suggestion below:

- Suggest to delete sentence referring to a study that is unpublished (reference 19). Please review author guidelines to referring to unpublished data

- [Henrietta Blinder, Franco Momoli, Julia Bokhaut, Vanessa Bacal, Reuben Goldberg, Dhenuka Radhakrishnan, Sherri L. Katz, Predictors of adherence to positive airway pressure therapy in children: a systematic review and meta-analysis, Sleep Medicine, Volume 69, 2020, Pages 19-33,

ISSN 1389-9457, https://doi.org/10.1016/j.sleep.2019.12.015].

- Bedi PK, DeHaan K, Ofosu D, Olmstead D, MacLean JE, Castro-Codesal M. Predictors of NIV-related adverse events in children using long-term noninvasive ventilation. Pediatr Pulmonol. 2023 Dec;58(12):3549-3559. doi: 10.1002/ppul.26689. Epub 2023 Sep 13. PMID: 37701936.

METHODS

I think it is problematic to refer to a previous study that is unpublished, as it does not provide the reader with sufficient information to critically appraise this study nor a reference to read about it. Consider deleting references to a previous study or wait until it is published.

Please add mention to ethics approval at the beginning of the methods section, including the name of committee that approved it and move information about required consents higher up in the methods section. It is currently quite further down, under a table, which keep the reader wonder what your intention is for a while.

Other comments to improve the methods section include:

- Spell out GOSH first time it is used.

- I miss a paragraph clearly describing inclusion/exclusion criteria. This is a good opportunity to describe who you are targeting (parents, children above certain age, neurocognitive status, staff members, reps, etc.) in each phase as well as specify your definition of NIV (CPAP, BPAP, mouth piece?) . There is no reference to Table 1 in the main text that I could find. I highly suggest to clearly write out the inclusion criteria and eliminate the table 1. Some of the information in the paragraphs below the table can be used to define the inclusion criteria and avoid redundancy.

- Suggest to add sampling matrix as a sentence separated by comas rather than an item list for better use of space. Please specify if that matrix applies to the children using NIV or their parents or both and how authors are planning to use it (i.e. are you considering recruiting at least one participant with each characteristics; are you planning to meet at least xx% of your matrix criteria?).

- About your matrix criteria: Are you considering gender or sex or both? Please specify rationale to your approach in the text. How are you going to determine level of adherence? Please specify.

- Consider rephrasing recruitment and sampling in phase 3. For instance, as it is right now, info in brackets is essential to understand your plan.

- Invitation approach needs some rephrasing. As it is right now, it is unclear to the reader if the NIV study member will introduce the study to the families at the end or beginning of a clinical visit or through an all user email. Please specify where email information will be found (I assume through a patient list).

-As per general comment above, please consider eliminating the use of item lists that make the text long and are not conventional in scientific writing. It can only be written in a tight sentence.

- Paragraph about recruitment and sample size. As suggested above, some of that information can be moved up to inclusion criteria and this paragraph limited to expected recruitment numbers. Justification of sample numbers based on previous studies requires references.

METHODS

PHASE 1 AND 2

1) Realistic review.

- Needs a reference to the methods used.

- Please add details of the systematic review methods including terms for search strategy (should be added as an appendix; if search strategy not designed yet, explain the reader how you are planning to develop the search: which kind of terms are you planning to use? who are you engaging to design your search strategy?. Please also add details on your screening process (double reviewer?) and extraction and analysis process.

- Can you clarify if you will be using PRISMA to report the results?

- Can you clarify how realistic review and workshop will be interacting to each other? As I read in the main text, looks like the search strategies will be presented in the workshops. However figure 2 shows it will inform stage 3 and 5, which is passed the search stage.

2) Workshops.

Can you clarify if you are planning to run separate workshops for children suing NIV, parents, staff members and manufacture representatives? As it reads right now, it sounds like everybody will be invited to participate together in a series of workshops? Also, can you clarify which languages you will be able to provide translation?

It might benefit the reader to have a one sentence explaining the behavior change wheel. Many readers might not be familiar with the concept.

I think the overall text can be synthesize and shorten to provide the essential information and allow for more detail on important information. For instance, in the paragraph about workshop 1 there is a reference to an adherence study that is not published or explained

through the manuscript. More detail is required for the reader to understand this sentence. Suggest to rephrase to a previously study done in our center analyzing .... As well, as per previous comments, it is unclear to me what part of the realistic review will be shared with participants. Is it the search strategy for the participants to contribute with the terms they think are relevant or are you sharing preliminary data extraction and synthesis?

3) Development of intervention toolkit. This paragraph mention a study steering committee that has not been mentioned before. If relevant, perhaps authors can describe who is part of this committee before. Apart from that, it mentions some examples of interventions that might come up but does not provide information on how these tools will be developed. This can be a significant amount of work and require a prolonged period of time. Can authors provide more information about this development phase?

Phase 3 paragraph. I find this paragraph lengthy and with too much level of detail. I suggest significant editing to reduce it and chose the essential information the reader requires to understand the study.

No reference to recruitment is provided. Previous paragraph about recruitment for phase 1 and 2 suggested recruitment of 8-10 patients/families. Does that apply to phase 3 too?

I struggle to understand how the patient/family-selected outcome will be measured and whether children and parents need to agree on the selected outcome to begin with. Would that be through a qualitative analysis of the interviews? Any other objective outcome measures other than actigraphy?

Key information about what the post-intervention questionnaire is lacking. Is this a validated questionnaire or will be designed ha doc for this study? Can you provide a reference?

Data analysis

It is hard to tease out if a framework-based analysis will be used for synthesis of both initial workshops as well as conversations/interviews during phase 3? Do you have an a priori idea of what framework you are planning to use? I imagine phase 1/2 and phase 3 will need different frameworks as they aim to collect different information.

In phase 3, can you provide a description to your plans to summarize data from the diaries?

My concerns of the quantitative analysis is that is not clear to me what the key variables are. It will help to develop a section with the specific study outcomes the authors are planning to analyze. I am concerned about the power of the study. There is no information on estimated sample size or recruitment expected # in phase 3.

Pages 16 to 18 are an example of a proposal-based format that do not meet manuscript scientific writing standards. Information about ethics approval should be moved up to beginning of the methods section and limited to one sentence. Please see author's guidelines for requirements of ethics specification. The table is not required.

Rather than dissemination plan, it would make more sense to have a short discussion paragraph discussing the anticipated importance of this study research.

Figure 3 does not provided added value. Suggest to delete.

All the best,

Reviewer **2**

Name **Yamauchi, Yusuke**

Affiliation **Aichi Children's Health and Medical Center, Department of Anesthesiology**

Date **22-Jan-2024**

COI **None**

This manuscript presents interesting and important findings and addresses the challenging issue of non-invasive ventilation (NIV) adherence for children. Although NIV has been shown to be effective for children with sleep disordered breathing (SDB), the major practical problem of adherence remains a concern for many healthcare providers and patient families. The authors successfully address this issue using a mixed methods model with Realist Review. However, I have a few comments and I hope this can contribute to a better manuscript.

Page 4 line 29: Limitation

There might be a selection bias in the sampling or invitation phase of this study. As the author wrote on Page9 line 7-19, the diversity of children is assured in the sampling, but there may be a selection bias because parents providing consent for the study are included in the sample. Please add to the limitation if necessary.

Page 4 line 29: Limitation

There may be a behavioral bias on this research. Children and young people (CYP) is assessed and observed in many ways. For example, Actiwatch is used to assess sleep quality. This assessment and observation may produce Hawthorne effect. [1] Furthermore, depending on the age and background of the CYP, there may be differences in their ability to recognize that they are being assessed and observed. Please add to the limitation if necessary.

Page 5 line 24: Reference 8 is a study showing predictors of chest infection in children with neuromuscular disease; additional literature is needed if it shows survival improvement with NIV.

Page 5 line 36: The reference is a study of children with neuromuscular disease. Thus, this sentence may be misleading in that it shows the effectiveness of NIV for a wide range of children.

Page 8 line: How would the authors clearly classify or define the level of adherence to NIV?

Page 13 line 35 – The authors will use a wrist Actiwatch for evaluating sleep quality. However, there have been reports of statistically significant differences in sleep assessment by Actiwatch when compared to polysomnography, which is commonly used for sleep assessment. How would you address the validity of the sleep assessment?

Reference

[1] Baddeley M. Herding, social influences and behavioural bias in scientific research: Simple awareness of the hidden pressures and beliefs that influence our thinking can help to preserve objectivity. EMBO Rep. 2015 Aug;16(8):902-5. doi: 10.15252/embr.201540637.

[2] Chinoy ED, Cuellar JA, Huwa KE, Jameson JT, Watson CH, Bessman SC, Hirsch DA, Cooper AD, Drummond SPA, Markwald RR. Performance of seven consumer sleep-tracking devices compared with polysomnography. Sleep. 2021 May 14;44(5):zsaa291. doi: 10.1093/sleep/zsaa291.

VERSION 1 - AUTHOR RESPONSE

Dear Mr Aldcroft

We would like to thank the reviewers for their constructive review of our protocol paper for the SPIRITUS study. We are grateful for the feedback and have detailed the changes that we have made as a result of the recommendations.

Dear Mr Aldcroft

We would like to thank the reviewers for their constructive review of our protocol paper for the SPIRITUS study. We are grateful for the feedback and have detailed below the changes that we have made as a result of the recommendations. Please note that we have changed the referencing system to using EndNote, so referencing will appear differently to the original manuscript.

Reviewer: 1
Dr. Maria Castro Codesal, University of Alberta, J.E MACLEAN
Comments to the Author:
Dear authors,

Thank you for putting together this important study. Please find my comments below to improve the clarity of your manuscript.

GENERAL COMMENTS

- The authors have written a thorough study protocol that is appropriate for a thesis proposal or grant application. However, further work is needed to adjust to scientific manuscript standards. Overall, it is a lengthy manuscript with significant amount of detail perhaps not always required while lacking some of the important information required to help the reader understand the purpose of the study and methods proposed. I have provided some examples below in comments to specific sections.

Thank you for bringing this to our attention. We've significantly reduced the Introduction and Data Collection sections.

- Eliminating item lists will improve the clarity of the manuscript and shorten it significantly.

All bullet point lists have been changed to sentence lists.

- Tables can also be eliminated and key information about inclusion criteria and ethics added to the manuscript in short paragraphs.

Information in tables regarding inclusion and ethics has been moved to the manuscript.

- It becomes sometimes redundant to have reference to phase 1, 2 and 3 several times through the text. I forces the reader to scroll up and down to find information about each phase in different parts of the manuscript. I wonder whether it would be more efficient use of the space and easier to read if authors create a section for each phase of the project that includes all pertinent information: realistic review (search strategy, screening process, data extraction and synthesis); workshops 1 & 2 (inclusion criteria, recruitment process, methods, data synthesis and analysis); and phase 3 (recruitment, data collection, and data analysis).

We agree with your comments regarding the order of information and we have now changed the sequence of information to make it easier for the reader to follow.

- I struggled with the acronyms used through the manuscript, some of them not spelled out on the first time they were used. There is a number of non-conventional acronyms (CYP, SSC, YPAG, CNS.) that are hard to memorize and retain in reader's mind through the manuscript reading. Consider revising all acronyms and only leave the essential ones.

We have removed all non-essential acronyms.

- There are a few format issues that might require authors to review author guidelines. For instance, abstracts and summaries do not include references, figures or tables.

We have removed the references and reference to the figures from the article summary.
- Authors have included references to unpublished studies and conference abstracts that do not meet authors guidelines.
These have now been removed.

SUMMARY:
Strengths section: I would argue that having a largest cohort is not a strength per se. Facilitation of adequate recruitment numbers or allowing for input from specialized healthcare providers with expertise in the use of NIV in pediatrics might. Having a large cohort can also be a limitation as it is because the study recruitment single center, as authors highlighted. Consider deleting it. The study have other methodological strengths including the high level of patient/family engagement and the use of multi-methods qualitative/quantitative that can be highlighted.

Thank you for these suggestions. We have amended the sentence regarding the size of the recruitment centre to: Our study site has one of the largest patient cohorts of paediatric NIV users in the UK, thereby allowing us to recruit sufficient numbers of children and families; recruitment will draw from a clinically heterogenous group which includes all the major condition groups where NIV use is likely.

- Spell out GOSH

We have spelt out the full name of the hospital.

- It is unclear to the reader what a traditional methods for data collection is. Suggest to rephrase towards highlighting innovate methods for qualitative data collection based on the use of new technologies.

We have changed this sentence to: ‘We will use a range of data collection methods, including digital and creative methods to create an inclusive research environment for a population that has a high rate of co-morbidities who may find traditional approaches alone hard to access.’

INTRO

- Sentence 'almost 1700 children in the United Kingdom (UK) use domiciliary NIV ' according to xxx [please provide the source].

We have amended this sentence to: ‘The child patient population is growing as indications for NIV expand In a recent scoping review NIV use was reported for 73 conditions; according to Wilkinson et al, almost 1700 children in the United Kingdom (UK) use domiciliary NIV.’

- SDB and OSA are used in the introduction, and it is unclear to the reader if they are interchangeable. Consider selecting one of the terms or describing how they are different. As well, it will help the reader to understand if NIV refers to CPAP and BPAP both from the introduction.

Thank you for drawing our attention to this. We have decided to remove this paragraph.

- References needs to be revised. For instance, sentence 'For these children there are few viable treatments to resolve their SDB : long-term NIV may be their only option (-needs a reference). CYP with sub-optimally treated SDB are at greater risk of chest infections and repeat hospitalizations (reference 14 only applies to patients with NMD and not to patients with OSA and other comorbidities).

We have removed this paragraph.

.- The last three paragraphs of the introduction could be more succinct and perhaps use more recent references.

These paragraphs have been significantly edited.

Suggestion below:

- Suggest to delete sentence referring to a study that is unpublished (reference 19). Please review author guidelines to referring to unpublished data

The reference has been amended.

- [Henrietta Blinder, Franco Momoli, Julia Bokhaut, Vanessa Bacal, Reuben Goldberg, Dhenuka Radhakrishnan, Sherri L. Katz, Predictors of adherence to positive airway pressure

therapy in children: a systematic review and meta-analysis, Sleep Medicine, Volume 69, 2020, Pages 19-33,
ISSN 1389-9457, https://doi.org/10.1016/j.sleep.2019.12.015].

- Bedi PK, DeHaan K, Ofosu D, Olmstead D, MacLean JE, Castro-Codesal M. Predictors of NIV-related adverse events in children using long-term noninvasive ventilation. Pediatr Pulmonol. 2023 Dec;58(12):3549-3559. doi: 10.1002/ppul.26689. Epub 2023 Sep 13. PMID: 37701936.

Both references have been included.

METHODS

I think it is problematic to refer to a previous study that is unpublished, as it does not provide the reader with sufficient information to critically appraise this study nor a reference to read about it. Consider deleting references to a previous study or wait until it is published.

References of published abstracts have been included.

Please add mention to ethics approval at the beginning of the methods section, including the name of committee that approved it and move information about required consents higher up in the methods section. It is currently quite further down, under a table, which keep the reader wonder what your intention is for a while.

The section on ethics approval has been moved.

Other comments to improve the methods section include:

- Spell out GOSH first time it is used.

GOSH has been spelled out.

- I miss a paragraph clearly describing inclusion/exclusion criteria.

We have taken this from the table and put it in the main body of text.

This is a good opportunity to describe who you are targeting (parents, children above certain age, neurocognitive status, staff members, reps, etc.) in each phase as well as specify your definition of NIV (CPAP, BPAP, mouth piece?) .

This definition has been added: “Non-invasive ventilation is defined as ventilatory assistance through a noninvasive interface, (i.e. not an endotracheal tube or a tracheostomy). It comprises continuous positive airway pressure (CPAP) and bilevel positive airway pressure, (BiPAP).”

We have also added information about participants for each phase of the study.

There is no reference to Table 1 in the main text that I could find. I highly suggest to clearly write out the inclusion criteria and eliminate the table 1. Some of the information in the paragraphs below the table can be used to define the inclusion criteria and avoid redundancy.

Table 1 has been deleted and the text incorporated into the manuscript.

- Suggest to add sampling matrix as a sentence separated by comas rather than an item list for better use of space. Please specify if that matrix applies to the children using NIV or their parents or both and how authors are planning to use it (i.e. are you considering recruiting at least one participant with each characteristics; are you planning to meet at least xx% of your matrix criteria?).

Details of the sampling matrix are now written in a sentence separated by commas and we have made it clear that the matrix applies to the children using NIV.

We have added in this sentence: “We will aim for an equal distribution of participation across these variables; to ensure parity we have set an upper and lower parameter for each variable in the matrix.”

- About your matrix criteria: Are you considering gender or sex or both? Please specify rationale to your approach in the text. How are you going to determine level of adherence? Please specify.

We have changed “gender” to “sex” and added in this sentence: “In situations where parents or children report that they or their child identifies differently to their birth assigned sex, we will take note of their preferred gender.”

In the absence of consensus in the literature as to how to define adherence, we will use the most common definition (Blinder H, Momoli F, Bokhaut J, Bacal V, Goldberg R, Radhakrishnan D, Katz SL. Predictors of adherence to positive airway pressure therapy in children: a systematic review and meta-analysis. Sleep Med. 2020 May;69:19-33. doi: 10.1016/j.sleep.2019.12.015.) Epub 2020 Jan 11. PMID: 32045851) with two categories of high adherence of usage that was >4 h use per night for >70% nights and low adherence for usage that was <4 h use per night for >70% nights).

- Consider rephrasing recruitment and sampling in phase 3. For instance, as it is right now, info in brackets is essential to understand your plan.

Recruitment and sampling have been rephrased.

- Invitation approach needs some rephrasing. As it is right now, it is unclear to the reader if the NIV study member will introduce the study to the families at the end or beginning of a clinical visit or through an all user email. Please specify where email information will be found (I assume through a patient list).

Invitation approach has been rephrased.

-As per general comment above, please consider eliminating the use of item lists that make the text long and are not conventional in scientific writing. It can only be written in a tight sentence.

All item lists have been removed.

- Paragraph about recruitment and sample size. As suggested above, some of that information can be moved up to inclusion criteria and this paragraph limited to expected recruitment numbers.

Recruitment and sampling have been rephrased.

Justification of sample numbers based on previous studies requires references.

References have now been added.

METHODS
PHASE 1 AND 2
1) Realistic review.
- Needs a reference to the methods used.
- Please add details of the systematic review methods including terms for search strategy (should be added as an appendix; if search strategy not designed yet, explain the reader how you are planning to develop the search: which kind of terms are you planning to use? who are you engaging to design your search strategy?. Please also add details on your screening process (double reviewer?) and extraction and analysis process.

Thank you for raising these important points. Details regarding the search strategy, screening process, extraction and analysis have been added. The reference for the Realist Review is: Pawson R, Greenhalgh T, Harvey G, Walshe K. Realist review--a new method of systematic review designed for complex policy interventions. J Health Serv Res Policy. 2005 Jul;10 Suppl 1:21-34. doi: 10.1258/1355819054308530. PMID: 16053581.

- Can you clarify if you will be using PRISMA to report the results?

We will use RAMESES to report the results as this reporting structure has been specifically designed for Realist Reviews.

- Can you clarify how realistic review and workshop will be interacting to each other? As I read in the main text, looks like the search strategies will be presented in the workshops. However figure 2 shows it will inform stage 3 and 5, which is passed the search stage.
We have revised the text to make it clearer that the results of the first literature searches will be presented in the first round of workshops and that any interventions identified in the workshops will inform iterative searches in the literature.

2) Workshops.
Can you clarify if you are planning to run separate workshops for children suing NIV, parents, staff members and manufacture representatives? As it reads right now, it sounds like everybody will be invited to participate together in a series of workshops?

We have amended this sentence to make it clearer: “Separate workshops will be held with children who use NIV and their parents, and staff groups across a range of disciplines (medical, nursing and allied health professionals) and representatives from industries that manufacture ventilator equipment for children.”

Also, can you clarify which languages you will be able to provide translation?

We have amended this sentence to make it clearer:

GOSH serves an ethnically diverse population and as such, it would not be possible to predict which languages we would need an interpreter for in advance. We will offer families the choice of either the research team providing an interpreter via the hospital interpreting services or the participant bringing a member of their community to act as an interpreter. We have amended this sentence:

“Interpreters will be available to facilitate the inclusion of non-English speaking children and parents on an as needed basis.”

It might benefit the reader to have a one sentence explaining the behavior change wheel. Many readers might not be familiar with the concept.

We have inserted this explanation: “This is a theoretical framework for understanding how behaviours change as a result of an intervention, identifying whether current and proposed interventions impact the three major domains of motivation, capacity and opportunity.”

I think the overall text can be synthesize and shorten to provide the essential information and allow for more detail on important information.

For instance, in the paragraph about workshop 1 there is a reference to an adherence study that is not published or explained through the manuscript. More detail is required for the reader to understand this sentence. Suggest to rephrase to a previously study done in our center analyzing ....

We have changed this sentence to: “In workshop 1, participants will be invited to discuss interventions that impact adherence and NIV outcomes from two sources: findings from a previously completed study at our centre and the results from the preliminary extraction and synthesis from the Realist Review, as well as any interventions that they have identified.”

As well, as per previous comments, it is unclear to me what part of the realistic review will be shared with participants. Is it the search strategy for the participants to contribute with the terms they think are relevant or are you sharing preliminary data extraction and synthesis?

We’ve clarified this sentence: “In workshop 1, participants will be invited to discuss interventions that impact adherence and NIV outcomes from two sources: findings from a previously completed study at our centre and the results from the preliminary extraction and synthesis from the Realist Review, as well as any interventions that they have identified.”

3) Development of intervention toolkit. This paragraph mention a study steering committee that has not been mentioned before. If relevant, perhaps authors can describe who is part of this committee before. Apart from that, it mentions some examples of interventions that might come up but does not provide information on how these tools will be developed. This can be a significant amount of work and require a prolonged period of time. Can authors

provide more information about this development phase?

We have added further detail about the study steering committee membership in the section about PPI. Further information has also been added about the tool development phase of the project.

Phase 3 paragraph. I find this paragraph lengthy and with too much level of detail. I suggest significant editing to reduce it and chose the essential information the reader requires to understand the study.

This paragraph has undergone additional editing to make the text more succinct.

No reference to recruitment is provided. Previous paragraph about recruitment for phase 1 and 2 suggested recruitment of 8-10 patients/families. Does that apply to phase 3 too?

We apologise for this omission and have added this information.

I struggle to understand how the patient/family-selected outcome will be measured and whether children and parents need to agree on the selected outcome to begin with. Would that be through a qualitative analysis of the interviews? Any other objective outcome measures other than actigraphy?

Measurement of the outcome will depend on what the outcome is – e.g. if the outcome is improved attendance at school we will measure attendance during the two weeks of the testing of the intervention and compare it with the two weeks prior to the intervention being tested. Similarly, if the outcome relates to sleep quality we may use a validated questionnaire. We have added further text about this. We will also collect ventilator download data as additional objective data.

Key information about what the post-intervention questionnaire is lacking. Is this a validated questionnaire or will be designed ha doc for this study? Can you provide a reference?

We recognise that the individualised approach presents a challenge in terms of measuring the selected outcome but the reviewer is correct in assuming that the questionnaire will be developed for this study. We have added in a further sentence outlining that questionnaires will be developed around the intervention and outcome chosen and will include issues related to feasibility, acceptability and impact of the intervention.

Data analysis

It is hard to tease out if a framework-based analysis will be used for synthesis of both initial workshops as well as conversations/interviews during phase 3? Do you have an a priori idea of what framework you are planning to use? I imagine phase 1/2 and phase 3 will need different frameworks as they aim to collect different information.

We have added in this sentence: “The Phase 3 framework will seek to identify participants’ (positive and negative) experiences of using the intervention during the two week period, how the parent and child engaged with it, what (if any) impact it had on their child’s NIV use and the wider family household, if the intervention could be changed in any way.”

In phase 3, can you provide a description to your plans to summarize data from the diaries?

We have added in this sentence: “Parent entries in their diaries will be used to inform both the post intervention debrief as well as analysed in their own right and included in the Phase 3 frameworks.”

My concerns of the quantitative analysis is that is not clear to me what the key variables are. It will help to develop a section with the specific study outcomes the authors are planning to analyze. I am concerned about the power of the study. There is no information on estimated sample size or recruitment expected # in phase 3.

We have added in detail about the sample size for phase 3. Phase 3 is designed to address feasibility and acceptability of the toolkit and is not powered to detect changes over time. We have clarified that the analysis will focus on feasibility and acceptability of the toolkit as well as data related to recruitment, retention and fidelity to the intervention.

Pages 16 to 18 are an example of a proposal-based format that do not meet manuscript scientific writing standards. Information about ethics approval should be moved up to beginning of the methods section and limited to one sentence. Please see author's guidelines for requirements of ethics specification. The table is not required.

We have moved these paragraphs in line with your feedback and added in information about our ethical procedures in line with the authors’ guidelines.

Rather than dissemination plan, it would make more sense to have a short discussion paragraph discussing the anticipated importance of this study research.

We have added in a short discussion on the anticipated importance alongside study outpts.

Figure 3 does not provided added value. Suggest to delete.

Done

All the best,
Thank you for providing such detailed feedback.

Reviewer: 2
Dr. Yusuke Yamauchi, Aichi Children's Health and Medical Center
Comments to the Author:
This manuscript presents interesting and important findings and addresses the challenging issue of non-invasive ventilation (NIV) adherence for children. Although NIV has been shown to be effective for children with sleep disordered breathing (SDB), the major practical problem of adherence remains a concern for many healthcare providers and patient families. The authors successfully address this issue using a mixed methods model with Realist Review. However, I have a few comments and I hope this can contribute to a better manuscript.

Page 4 line 29: Limitation
There might be a selection bias in the sampling or invitation phase of this study. As the author wrote on Page9 line 7-19, the diversity of children is assured in the sampling, but there may be a selection bias because parents providing consent for the study are included in the sample. Please add to the limitation if necessary.
We have added this as a further limitation.

Page 4 line 29: Limitation
There may be a behavioral bias on this research. Children and young people (CYP) is assessed and observed in many ways. For example, Actiwatch is used to assess sleep quality. This assessment and observation may produce Hawthorne effect. [1] Furthermore, depending on the age and background of the CYP, there may be differences in their ability to recognize that they are being assessed and observed. Please add to the limitation if necessary.

Thank you for drawing our attention to this. In our study there will be no direct observation during day or night-time, either in a hospital or a home setting. The family will know that the researcher will collect the ventilator and Actiwatch data at the end of the study period. This

could potentially result in increased ventilator use. If their ventilator use does increase in the trial period, we will not interpret that finding in isolation as evidence of the success of the intervention and would also look at other forms of data in conjunction, such as the parent and child debrief.

Page 5 line 24: Reference 8 is a study showing predictors of chest infection in children with neuromuscular disease; additional literature is needed if it shows survival improvement with NIV.
Thank you for drawing our attention to this. We have decided to remove this paragraph.

Page 5 line 36: The reference is a study of children with neuromuscular disease. Thus, this sentence may be misleading in that it shows the effectiveness of NIV for a wide range of children.
Thank you – we have removed this paragraph.

Page 8 line: How would the authors clearly classify or define the level of adherence to NIV?

This is an important debate that lacks consensus in the literature. We've added in this sentence:

In the absence of consensus in the literature as to how to define adherence, we will use the most common definition (Blinder H, Momoli F, Bokhaut J, Bacal V, Goldberg R, Radhakrishnan D, Katz SL. Predictors of adherence to positive airway pressure therapy in children: a systematic review and meta-analysis. Sleep Med. 2020 May;69:19-33. doi: 10.1016/j.sleep.2019.12.015.) Epub 2020 Jan 11. PMID: 32045851) with two categories of high adherence of usage that was >4 h use per night for >70% nights and low adherence for usage that was <4 h use per night for >70% nights).

Page 13 line 35 – The authors will use a wrist Actiwatch for evaluating sleep quality. However, there have been reports of statistically significant differences in sleep assessment by Actiwatch when compared to polysomnography, which is commonly used for sleep assessment. How would you address the validity of the sleep assessment?
We've given this a lot of thought and spent time discussing this a research team. The variables we are collecting are around usage (number of hours of ventilation used, number of nights used and whether ventilation occurred during sleep). As we're measuring feasibility and acceptability we aren't going to collect any measurements around efficacy (i.e. to see if the intervention improves adherence and subsequently any of the standard measurements of gas change). We have increased the level of detail around measurement in these paragraphs:

Actiwatch data in conjunction with the ventilator will enable us to identify whether the child: 1) used their NIV during the two week period and 2) if they used their NIV when asleep. This will mitigate potential response bias, e.g. if parents report that the intervention resulted in complete adherence and the ventilator data show that the ventilator was not used in the study period. These data will also be collected to assess whether the intervention has caused a decrease in adherence.

We will compare usage data (number of hours used per night and number of nights used) during the 2 week trial period and usage data from the four weeks prior to the most recent ventilator download (usually in the NIV clinic or at the most recent sleep study).

Reference

[1] Baddeley M. Herding, social influences and behavioural bias in scientific research: Simple awareness of the hidden pressures and beliefs that influence our thinking can help to preserve objectivity. EMBO Rep. 2015 Aug;16(8):902-5. doi: 10.15252/embr.201540637.

[2] Chinoy ED, Cuellar JA, Huwa KE, Jameson JT, Watson CH, Bessman SC, Hirsch DA, Cooper AD, Drummond SPA, Markwald RR. Performance of seven consumer sleep-tracking devices compared with polysomnography. Sleep. 2021 May 14;44(5):zsaa291. doi: 10.1093/sleep/zsaa291.

We believe that the suggested revisions have improved our manuscript and we hope that it is now suitable for publication in *BMJ Open.*
